# A new resource for developing and strengthening large-scale community health worker programs

**DOI:** 10.1186/s12960-016-0178-8

**Published:** 2017-01-12

**Authors:** Henry Perry, Lauren Crigler, Simon Lewin, Claire Glenton, Karen LeBan, Steve Hodgins

**Affiliations:** 1 Department of International Health, Johns Hopkins Bloomberg School of Public Health, Baltimore, MD United States of America; 2 Crigler Global Consulting, Durham, NC United States of America; 3 Global Health Unit, Norwegian Knowledge Centre for the Health Services at the Norwegian Institute of Public Health, Oslo, Norway; 4 Health Systems Research Unit, Medical Research Council of South Africa, Cape Town, South Africa; 5 The CORE Group, Washington, DC United States of America; 6 Saving Newborn Lives/Save the Children, Washington, DC United States of America

**Keywords:** Community health workers, Primary health care, Community health, Community-based primary health care

## Abstract

Large-scale community health worker programs are now growing in importance around the world in response to the resurgence of interest and growing evidence of the importance of community-based primary health care for improving the health of populations in resource-constrained, high-mortality settings. These programs, because of their scale and operational challenges, merit special consideration by the global health community, national policy-makers, and program implementers. A new online resource is now available to assist in that effort: *Developing and Strengthening Community Health Worker Programs at Scale: A Reference Guide and Case Studies for Program Managers and Policymakers* (http://www.mchip.net/CHWReferenceGuide). This CHW Reference Guide is the product of 27 different collaborators who, collectively, have a formidable breadth and depth of experience and knowledge about CHW programming around the world. It provides a thoughtful discussion about the many operational issues that large-scale CHW programs need to address as they undergo the process of development, expansion or strengthening. Detailed case studies of 12 national CHW programs are included in the Appendix—the most current and complete cases studies as a group that are currently available. Future articles in this journal will highlight many of the themes in the CHW Reference Guide and provide an update of recent advances and experiences. These articles will serve, we hope, to (1) increase awareness about the CHW Reference Guide and its usefulness and (2) connect a broader audience to the critical importance of strengthening large-scale CHW programs for the health benefits that they can bring to underserved populations around the world.

## Background

### Commentary

The rapid growth of interest in and development of community or lay health worker (CHW) programs in low-income settings over the past decade [1–3] is a welcome development for so many of us who have long seen the potential for stronger community-based programs to improve the health of populations and particularly the health of mothers and children. There is growing evidence that impressive gains can be made in smaller populations with well-trained and well-supported CHWs implementing discrete interventions over a relative short period of time, including interventions for health promotion and for the prevention and treatment of serious conditions that are leading causes of mortality [4, 5]. Furthermore, a substantial number of countries with strong, large-scale CHW programs—for example, Bangladesh [6, 7], Brazil [7], Ethiopia [7–10], Malawi [11], and Nepal [7]—have made remarkable progress in expanding the coverage of key maternal and child health interventions. These countries have shown impressive gains in reducing maternal and child mortality and in expanding the coverage of family planning services with concomitant reductions in fertility.

The resurgence in CHW programming has been slow in coming. An important initial upswing of enthusiasm and experience with large-scale CHW programs in the late 1970s and early 1980s was associated with their endorsement at the International Conference on Primary Health Care and in the Declaration of Alma-Ata [12]. However, this was followed in the later 1980s by the implosion of many of these programs, disillusionment with the approach, and an increased focus on vertical programs for the delivery of key health interventions [13–15].

There were many reasons for this turn of events—from politics to the global economic recession of the 1980s to the influence of donors with a strong technical, vertical approach for improving population health. Also important was that the planning and scale-up of programs was often hurried, there was not a strong appreciation of the critical need for strong supervision and logistical support, and the cost required for effective functioning was underestimated. In addition, monitoring and evaluation of these programs was lacking, and there was a lack of strong commitment to the use of monitoring and evaluation findings for program strengthening.

### A new reference guide

A recently released reference guide [16], drawing on a number of case studies of large-scale CHW programs, attempts to address some of these earlier challenges by providing a thoughtful discussion about the structure and function of such CHW programs. This guide is intended to assist policy-makers, planners, and program implementers in strengthening existing large-scale programs and in designing and scaling up new programs. Entitled, *Developing and Strengthening Community Health Worker Programs at Scale: A Reference Guide and Case Studies for Program Managers and Policymakers*, it is the product of 27 different collaborators who, collectively, have a formidable breadth and depth of experience and knowledge about CHW programming around the world. It was made possible by funding from USAID, through the MCHIP project.

The CHW Reference Guide, as we refer to it, is available online at http://www.mchip.net/CHWReferenceGuide, and can be downloaded in its entirety of 468 pages or chapter by chapter. It contains chapters in four main sections: (1) Setting the Stage (with chapters on history of CHW programs, planning, governance, financing, and national coordination and partnerships), (2) Human Resources (roles and tasks, recruitment, training, supervision, and incentives), (3) CHW Programs in Context (relationships with other parts of the health system, and relationships with the community), and (4) Achieving Impact (scaling up and sustainability, and measurement and data use). An extensive appendix contains case studies, perspectives from key informants, and important resources. There are case studies of national CHW programs in 12 different countries (Afghanistan, Bangladesh, Brazil, Ethiopia, India, Indonesia, Iran, Nepal, Pakistan, Rwanda, Zambia, and Zimbabwe). As a group, these case studies are the most current and complete descriptions of these programs available. The appendix also contains a summary of key informant interviews that provide important insights into challenges that large-scale CHW programs face.

The Reference Guide addresses issues and challenges that all large-scale CHW programs face, and it offers many examples of how specific programs have addressed these issues. It does not try to present a simple (or a single) solution, but rather raises questions that need to be considered by policy-makers and program implementers in their own particular context, along with possible options and resources for addressing these questions. The Guide does *not* address specific technical issues related to specific interventions (such as the range of interventions that CHW can provide, the training and logistical support required for specific interventions, and so forth).

Over the next few months, *Human Resources for Health* will be publishing a series of papers that are derived from the CHW Reference Guide and that address in more critical detail broad issues facing large-scale CHW programs. Our hope is that these articles will serve to highlight some of the important issues addressed in the CHW Reference Guide and to raise further interest in the use of the Guide itself.

## Conclusion

The recently released CHW Reference Guide is an important resource for policy-makers, planners, and program implementers in strengthening existing large-scale programs and in designing and scaling up new programs. This resource and forthcoming articles in *Human Resources for Health* will provide much needed support to the important task ahead of making large-scale national CHW programs as effective as possible.

## Abbreviations

CHW: Community health worker
MCHIP: Maternal and Child Health Integrated Program
USAID: United States Agency for International Development
